# Screening for diabetic retinopathy using new mydriasis-free, full-field flicker ERG recording device

**DOI:** 10.1038/srep36591

**Published:** 2016-11-08

**Authors:** Motonobu Fukuo, Mineo Kondo, Akira Hirose, Harumi Fukushima, Kengo Ikesugi, Masahiko Sugimoto, Kumiko Kato, Yasuko Uchigata, Shigehiko Kitano

**Affiliations:** 1 Department of Diabetic Ophthalmology, Diabetes Center, Tokyo Women’s Medical University, Tokyo, Japan; 2 Department of Ophthalmology, Mie University Graduate School of Medicine, Tsu, Japan; 3 Diabetes Center, Tokyo Women’s Medical University School of Medicine, Tokyo, Japan

## Abstract

Diabetic retinopathy (DR) is a leading cause of blindness among working-age adults. Therefore, it is important to detect DR accurately during mass screening. The purpose of this study was to determine whether a small, hand-held, mydriasis-free, full-field flicker electroretinographic (ERGs) device called RET*eval* can be used to screen for DR. To accomplish this, we recorded full-field flicker ERGs with this device from 48 normal eyes and 118 eyes with different severities of DR in patients with diabetes mellitus (DM). This system delivered a constant flash retinal luminance by adjusting the flash luminance that compensated for changes in the pupil size. Our results showed that there were significant correlations between the severity of DR and the implicit times (*P* < 0.001; *r* = 0.55) and the amplitudes (*P* = 0.001; *r* = −0.29). When the implicit time was used for the index, the area under the receiver operating characteristic curve was 0.84 for the detection of DR, and was 0.89 for the detection of DR requiring ophthalmic treatments. These results suggest that the implicit times of the flicker ERGs recorded by the small, mydryasis-free ERG system can be used as an adjunctive tool to screen for DR.

Diabetic retinopathy (DR) is the most common cause of vision reduction among individuals with diabetes and a leading cause of blindness among working-age adults^1^,^2^,^3^,^4^. Therefore, it is important to detect and classify DR accurately during mass screening. For this, a variety of ophthalmological tests have been used including direct and indirect ophthalmoscopy, stereoscopic color fundus photography, and mydriatic or nonmydriatic digital color or monochromatic photography^5^. Although stereoscopic color fundus photography in 7-standard fields is still the gold standard to screen for DR^6^, this technique requires skilled photographers and analyzers and is uncomfortable and time consuming for the individuals^7^. On the other hand, a single-field non-mydriatic fundus photograph is relatively easy to do, but the limitations include a higher technical failure and lower sensitivity than the 7-standard field photographs^8^,^9^,^10^.

Full-field electroretinography (ERG) is a non-invasive physiological test^11^, and it has been widely used for the objective assessment of retinal function including eyes with DR^12^,^13^. Although it is difficult to detect diabetic macular oedema (DMO) by full-field ERGs because this is a full-field retinal functional test, earlier studies have shown that one of the first signs of DR is a prolongation of the implicit time or a reduction of the amplitude of the oscillatory potentials of the full-field ERGs^14^,^15^,^16^. Other studies also showed that the implicit times of the photopic ERG b-waves^17^,^18^ or the 30-Hz flicker ERGs^18^,^19^,^20^,^21^ were highly correlated with the severity of the DR. Conventional ERG recordings have not been considered as a method for the mass screening of DR because the ERG recordings requires a large space for the recording system and long times to collect meaningful data.

Recently, a new, small, full-field ERG recording device called the RET*eval* system was introduced. This device is equipped with a small Ganzfeld dome and a special skin electrode to pick up the ERGs^22^,^23^,^24^,^25^. The full-field ERGs can be recorded without mydriasis because this device delivers stimuli of constant retinal illuminance (Td-s) by adjusting the luminance (cd-s/m^2^) to compensate for changes in the pupillary area (mm^2^). The total ERG recording time is within 60 seconds if only the flicker ERGs are recorded. Thus, the application of this new device for the mass-screening for DR has been suggested. However as best we know, there has been only one study^24^ that examined whether the RET*eval* system can be used to screen individuals for the presence of DR. Maa *et al*.^24^ studied the performance of RET*eval* device that uses a combination of flicker ERG and pupillography, and reported that the RET*eval* device can identify the patients with diabetes mellitus (DM) as not having vision-threatening diabetic retinopathy with 99% accuracy. However, as best we know, it has not been determined whether the RET*eval* device can be used to identify patients with DR.

Thus, the purpose of this study was to determine whether the RET*eval* system can be used for the mass-screening of eyes that have DR. To accomplish this, we recorded full-field flicker ERGs using the RET*eval* device from normal eyes and eyes with different severities of DR.

## Results

The demographics for the 48 normal subjects and 118 patients with DM are shown in Table 1. There was no significant difference in the age between all six groups. There was also no significant difference in HbA1c level between the five groups of patients with the different severities of DR. On the other hand, there were significant differences in the duration of DM for the five groups of patients with DM (*P* < 0.001). Patients with the more severe DR tended to have longer duration DM. There were also significant differences in the best-corrected visual acuity (BCVA) between the six groups (*P* < 0.001). Patients with the more severe stages of DR tended to have worse BCVA.

The prevalence of DR, DR requiring ophthalmologic therapy, viz., vision-threatening diabetic retinopathy, and DMO in our DM cohort were 37.2%, 16.9%, and 7.6%, respectively. These prevalences are approximately the similar to that of the global DM population^26^.

**Figure 1:**
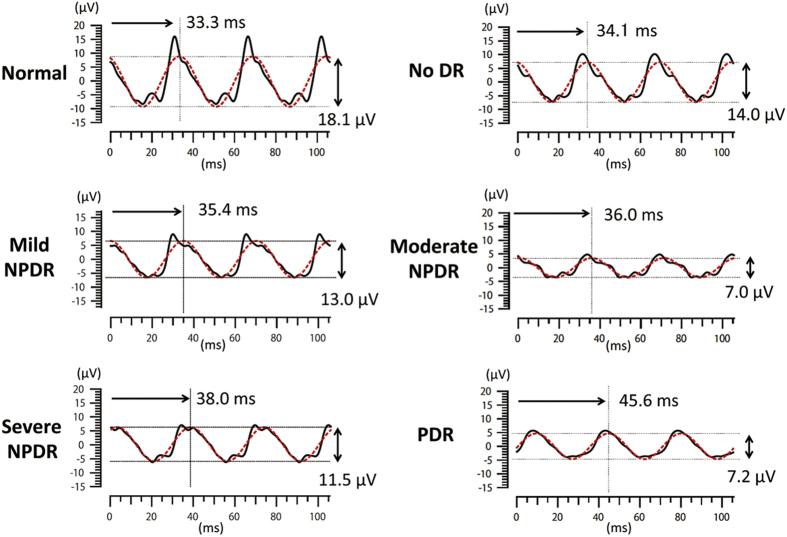
Representative flicker ERGs recorded from a normal eye and five eyes with diabetes mellitus (DM) with various severities of diabetic retinopathy (DR) including no DR, mild nonproliferative DR (NPDR), moderate NPDR, severe NPDR, and proliferative DR (PDR). The solid black line indicates the “whole waveform”, viz., the reconstructed waveform using the first eight harmonics, of the flicker ERGs, and the red dotted line represents the fundamental component. The actual values of the amplitudes and implicit times of the fundamental components are also shown in the figure.

Representative flicker ERGs recorded from a normal eye and five eyes with different severities of DR are shown in Fig. 1. The solid black line indicates the “whole waveform”, viz., the reconstructed waveform using the first eight harmonics of the flicker ERGs, and the red dotted line represents the fundamental component. The actual values of the amplitudes and implicit times of the fundamental components are also shown in the figure. In general, the implicit times were longer and the amplitudes were lower with more severe DR.

**Figure 2:**
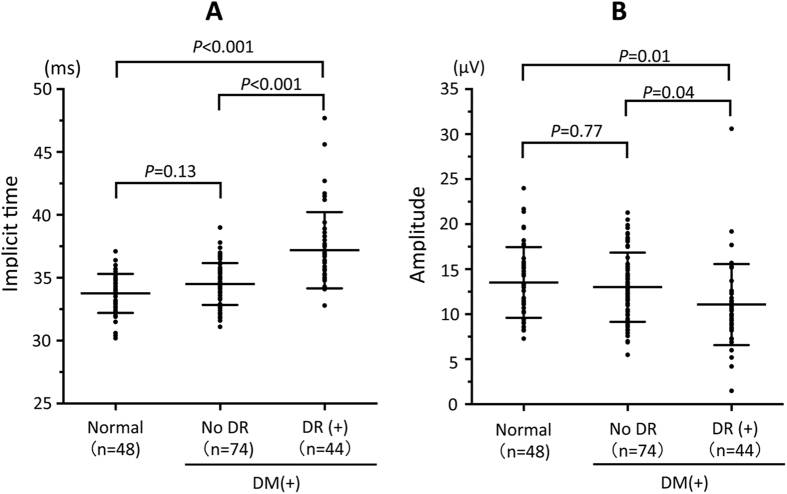
Implicit times (**A**) and amplitudes (**B**) of fundamental component of flicker ERG for normal eyes (n = 48), DM eyes without DR (no DR, n = 74), and DM eyes with DR (=DR (+), n = 44). Long and short horizontal bars in the plots indicate the mean and standard deviation, respectively.

All of the 166 eyes were divided into three groups: normal eyes (n = 48), eyes with no DR (n = 74), and eyes with DR (n = 44). The implicit times and the amplitudes of the fundamental component of the flicker ERG recorded with the RET*eval* device in the three groups were compared to determine whether they were any significant differences (Fig. 2). The results indicated that there were no significant differences in the implicit times and amplitudes between the normal eyes and eyes with no DR. On the other hand, the implicit times in eyes with DR were significantly longer than that of normal eyes and eyes with no DR (*P* < 0.001). The amplitude in eyes with DR was also significantly smaller than the amplitude in normal eyes (*P* = 0.01) and eyes with no DR (*P* = 0.04).

**Figure 3:**
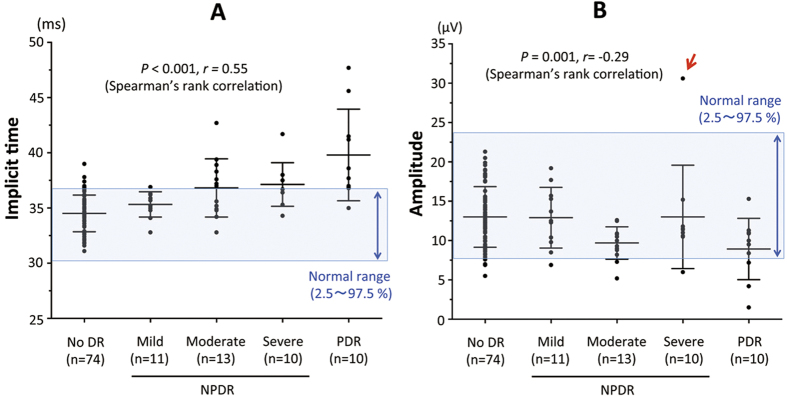
Implicit times (**A**) and amplitudes (**B**) of fundamental component of flicker ERGs for DM eyes without DR (=no DR, n = 74), mild NPDR (n = 11), moderate NPDR (n = 13), severe NPDR (n = 10), and PDR (n = 10). Long and short horizontal bars in the plots represent the means and standard deviations, respectively. The areas of light blue indicate normal range, which was determined by 2.5–97.5 percentile of normal eyes.

Next, we examined whether there were significant correlations between the severity of the DR and the implicit times and the amplitudes of the flicker ERGs recorded by RET*eval* in the 118 eyes with DM (Fig. 3). Our results indicated that there was a significant positive correlation between the severity of DR and the implicit times (*P* < 0.001) with the implicit times more delayed with more severe DR (Fig. 3A). The amplitude was also significantly and negatively correlated with the severity of the DR (*P* = 0.001), and the amplitude was smaller with more severe DR (Fig. 3B). The correlation between the severity of the DR was weaker with the amplitude (*r* = −0.29) than with the implicit time (*r* = 0.55). Interestingly, the mean amplitude in eyes with severe NPDR was greater than that in eyes with moderate NPDR (*P* = 0.045). The amplitude of one eye with severe NPDR was greater than the upper limit of the normal range (red arrow, Fig. 3B).

**Figure 4:**
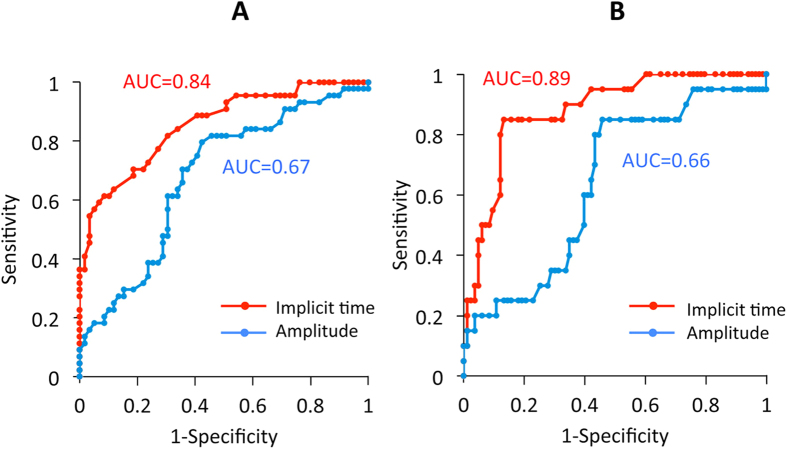
(**A**) Receiver operating characteristic (ROC) curve for the detection of DR (mild NPDR, moderate NPDR, severe NPDR and PDR). (**B**) ROC curve for the detection of DR requiring ophthalmic treatments (severe NPDR and PDR). Red and blue lines show the implicit time and amplitude of fundamental component of flicker ERG used as an index.

Finally, we studied whether the implicit times or the amplitudes of the flicker ERGs recorded with the RET*eval* can be used to classify the eyes into those with no DR (n = 122, normal eyes and eyes with no DR) and those with DR (n = 44, eyes with mild, moderate, or severe NPDR and eyes with PDR) using the ROC curve. We found that the AUC was 0.84 when the implicit times were used and 0.67 when the amplitudes were used (Fig. 4A). When the implicit time was used, the optimal cut-off point was 35.6 ms, resulting in a sensitivity of 0.70 and a specificity of 0.81.

Similarly, we studied whether the implicit time or the amplitude of the flicker ERG recorded by RET*eval* would facilitate the classification of eyes into those with DR not requiring ophthalmologic therapy (n = 122, normal eyes, eyes with no DR, eyes with mild NPDR, and eyes with moderate NPDR) and those with DR requiring ophthalmologic therapy (n = 20, eyes with severe NPDR and PDR) using the ROC curve. The results indicated that the AUC was 0.89 when the implicit times were used and 0.66 when the amplitudes were used (Fig. 4B). When the implicit times were used, the optimal cut-off point was 36.4 ms, resulting in a sensitivity of 0.85 and a specificity of 0.85. Assuming that the prevalence of DR requiring ophthalmologic therapy is 0.90% in general population ages ≥20 years^26^,^27^, the positive and negative predictive values of the implicit time were estimated to be 4.8% and 99.8%, respectively.

## Discussion

The results demonstrated that the implicit time of the flicker ERGs recorded and analyzed by the RET*eval* system was more highly correlated with the severity of the DR than the amplitude (Fig. 3). In addition, the implicit times had higher AUCs of the ROC curve for the detection of both DR and DR requiring ophthalmological therapy (Fig. 4). These findings suggest that the implicit time of the flicker ERG recorded by RET*eval* can be used for the screening of DR.

Many past studies have reported that the ERGs can be a useful test in screening for DR^12^,^13^,^14^,^15^,^16^,^17^,^18^,^19^,^20^,^21^, but there have been few instances when ERGs were used for screening for DR. There are three major reasons for this; a large space is required for the conventional equipments to record ERGs, mydriasis and topical anesthesia are needed prior to ERG recordings, and inserting the electrodes for ERG recordings can be difficult and somewhat traumatic. The RET*eval* is a small portable device that can record the flicker ERG without mydriasis. In addition, this device uses a single skin adhesive-tape electrode, which can be attached without anesthesia. If the examiner wants to record only the flicker ERGs, the total recording time including the preparation is less than one minute. Thus, we conclude that the RET*eval* device was more suitable for mass screening than the conventional ERG recordings systems.

One interesting phenomenon seen in the present study was the relatively large amplitude flicker ERGs in the eyes with severe NPDR (Fig. 3B). The mean amplitude of ten eyes with severe NPDR was significantly larger than that in ten eyes with moderate NPDR, and the amplitude of one eye with severe NPDR was larger than upper-limit of the normal range (red arrow, Fig. 3B). The exact reason why the eyes with severe NPDR tended to have larger amplitudes was not determined, but several past studies have shown that the retinas with some degrees of ischemia^28^,^29^,^30^,^31^,^32^,^33^,^34^ or hyperglycemia^35^ can have supernormal full-field ERG amplitudes. The changes in the retinal circulation or neuronal activity due to the DR may be responsible for the relatively larger amplitudes in the eyes with severe NPDR. Based on these findings combined with lower AUCs for the amplitudes, the amplitude of the flicker ERG may not be an appropriate index for the screening of DR.

**Figure 5:**
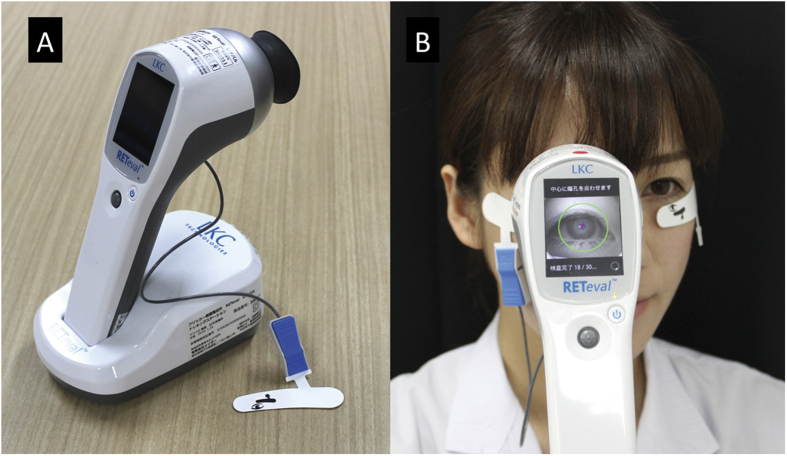
Photographs of the RET*eval* ERG recording system used in this study. (**A**) This system consists of a hand-held stimulator, recording and analysis system, a docking station for charging and downloading the results to a computer, and disposable skin electrode array. (**B**) Recording of full-field flicker ERG with the RET*eval* system. During the recording, the subject’s pupillary area is automatically measured by a built-in automated pupillometer, and a constant flash retinal illuminance (Td-s) is delivered.

The results showed that the implicit times of the flicker ERGs were correlated with the presence and severity of the DR, but we believed that it still cannot replace fundus examinations or fundus photographs based on three findings; the implicit time was within the normal range in one of ten eyes with PDR (Fig. 3A), the correlation between the severity of DR and implicit time was not so high (*r* = 0.55, Fig. 3A), and the AUC of the ROC curve was only a moderate value for the detection of DR (Fig. 5). These differences between the two tests are not surprising because the ERG is a physiological full-field retinal function test whereas the fundus examination or fundus photographs are assessment of the images. It is known that there are some eyes with DR in which the fundus findings appear relatively mild at the posterior pole but the retinal ischemia is very severe in the periphery. The implicit times of the full-field flicker ERGs may be especially useful in identifying such DR eyes with poor fundus findings in the periphery. These findings can be confirmed by wide-field imaging, fluorescein angiography, and optical coherence tomography. Thus, the implicit time of flicker ERG may be a more useful index to compensate for the drawbacks of fundus examinations.

Maa *et al*. used the RET*eval* device to screen for DR, and reported that it detected vision-threatening DR, e.g., severe NPDR or PDR, with a high level of sensitivity (AUC, 0.86)^24^. Their findings and the value of the AUC are comparable to our results. However, there are two major differences in the methods of testing between their study and our study. First, Maa *et al*. used not only the flicker ERGs but also the pupillary light responses as the indices to detect DR. Second, their stimulus intensity to elicit the flicker ERG was 32 photopic Td-s which is 0.6 log units higher than that in our study of 8 photopic Td-s. Thus, determining the optimal stimulus and analysis methods should be further studied so that the RET*eval* can be used more effectively in detecting DR.

This study has four limitations. The first is the small sample size of 48 normal eyes and 118 eyes with DR. Unfortunately, we could not obtain more eyes with the four stages of DR because we excluded previously treated eyes. Another study (Maa *et al*.^24^) had a more extensive design and a larger number of subjects. The larger number of subjects were randomized to separate calibration and validation groups. Thus, a different group of subjects was used to validate the parameters for detecting vision-threatening DR. Such an approach might have been possible in our study if a larger sample size had been available.

The second limitation is that the optimal stimulus settings of the RET*eval* device have not been determined for the detection of DR. We used a fixed stimulus flash retinal illuminance of 8 photopic Td-s which is about 1.3 log lower than the ISCEV standard^11^ for flicker stimulation. This is simply because this stimulus setting was the device’s default settings for flicker ERG for non-dilated eyes. However, the optimal stumulus conditions for the flicker ERG for the screening of DR need to be further studied.

The third limitation is that only the fundamental component of the flicker ERG, which is displayed automatically by the current RET*eval* system, was analyzed. However, it would be interesting to also analyze the full raw or reconstructed waveforms using the peak amplitudes and times from the averaged waveform. We are currently planning to compare the fundamental component and raw waveform to clarify which can detect DR more effectively in the next study.

The fourth limitation is that we excluded eyes with advanced cataract. In the real-world situation, the eyes with advanced cataract are common in patients with DM. Thus, the effects of cataract on the flicker ERG recorded by RET*eval*^25^ should be investigated.

In conclusion, we found that the RET*eval*, a small, non-mydriatic flicker ERG recording device, can be used as an adjunct tool to screen for DR. Further studies are needed to determine the optimal stimulus and analysis conditions using this device for the screening of DR.

## Methods

### Study Design

This was a prospective, cross sectional, single-center study conducted in accordance with the Institutional Guidelines of Tokyo Women’s Medical University and was approved by the Institutional Ethics Review Board (number, #3125). The procedures conformed to the tenets of the World Medical Association’s Declaration of Helsinki, and a written informed consent was obtained from all subjects after they were provided with sufficient information on the procedures to be used.

### Subjects

One hundred and eighteen eyes of 118 patients with DM and 48 eyes from 48 normal subjects were studied. The results of only the right eye were used for the statistical analyses. The patients with any other eye diseases including glaucoma were excluded. We graded the density of the lens opacity using the slit-lamp findings and the WHO cataract grading system^36^. Nuclear or subcapsular cataracts with grade 3 or more were excluded. The patients who had received any treatments for DR were also excluded. All of the normal subjects did not have any ocular or systemic disease. Subjects with myopia greater than −6 diopters were excluded from both the normal and DM groups.

### Ophthalmic Examinations

All of the patients with DM had comprehensive ocular examinations including measurements of the best-corrected visual acuity (BCVA), refractive error by autorefractometry, and intraocular pressure (IOP) with a non-contact tonometer. In addition, they had anterior segment examination by slit-lamp biomicroscopy. After mydriasis, fundus examinations were performed by indirect ophthalmoscopy, and color fundus photographs were recorded.

Based on the fundus examination by indirect ophthalmoscopy by three DR specialist [SK, AH, and HF], the severity of DR was classified into five categories: no DR, mild non-proliferative DR (mild NPDR), moderate NPDR, severe NPDR, and proliferative DR (PDR), according to the International Clinical Diabetic Retinopathy Disease Severity Scale^37^,^38^.

### Non-Mydriatic Flicker ERG Recordings with RET*eval* Device

The details of the RET*eval* (LKC Technologies, Gaithersburg, MD) device, a small, hand-held, non-mydriatic full-field flicker ERG recording system, have been described in detail^22^,^23^,^24^,^25^. The full-field flash stimuli are presented with a small dome of 60 mm in diameter (Fig. 1A). Visible “white” stimuli (CIE 1931 chromaticity, x = 0.33, y = 0.33) were presented by a combination of three colored light emitting diodes (LEDs; red, 622 nm; green, 530 nm; blue, 470 nm). The frequency of the flicker stimuli was 28.306 Hz and the pulse duration was less than 1 msec. A small red fixation spot is present at the center of the dome.

During the flicker stimulation, the pupil size (mm^2^) is automatically measured in real time, and the stimulus flash luminance (cd-s/m^2^) is continuously adjusted to keep a constant flash retinal illuminance (Td-s) according to the following equation: Photopic flash retinal illuminance (Td-s) = photopic flash luminance (cd-s/m^2^) x pupillary area (mm^2^).

The ERG signals were picked up by a special skin electrode array (Sensor Strip, LKC Technologies, Inc.) which was placed on the orbital rim of the lower eyelid (Fig. 1B). This electrode array contains three electrodes, an active (positive), a reference (negative), and a ground in a single adhesive tape. The electrical potentials are DC-amplified and digitized (sampling rate, 2 kHz).

We used a fixed stimulus flash retinal illuminance of 8 photopic Td-s, which is the recommended default stimulus setting for flicker ERGs for non-dilated eyes in the RET*eval* system. This stimulus intensity, 8 Td-s, is about 1.3 log lower than the ISCEV standard for flicker stimulation of 150 Td-s^11^. No background illumination was used. The recording time ranged from 5 to 15 seconds depending on the reliability of the results. In the end, the ERGs elicited by 141 to 425 flashes were analyzed for each recording.

The amplitudes and implicit times of the fundamental component were automatically measured and displayed by the RET*eval* system using a special algorithm of discrete Fourier transformation (DFT) and cross-correlation analysis^23^,^24^,^39^. In this device, two flicker ERG waveforms, the fundamental component and the reconstructed “whole” flicker ERG waveform using the first eight harmonics, are presented.

### Statistical Analyses

A one-way layout analysis of variance (ANOVA) was performed to examine the homogeneity of the background factors in each group. A one-way ANOVA with Tukey-type multiple comparison was used to compare the implicit times or amplitudes among the three groups of normal, no DR, and DR (+). Spearman’s rank correlation coefficient was used to examine if the correlations between the severity of DR and the implicit time or amplitude of the fundamental component of flicker ERGs were significant. The area under the curve (AUC) of the receiver operating characteristic (ROC) curve was used to evaluate the detection performance, the sensitivity and specificity, of the implicit times or amplitudes of the flicker ERGs to discriminate DR, or DR requiring ophthalmological treatments (severe NPDR or PDR). The cut-off values balancing sensitivity and specificity were determined by using the Youden index which was calculated as (sensitivity + specificity) −1. The positive and negative predictive values were estimated based on the global prevalence data of DM^27^ and DR^26^ in general population of 20 years or older. The results were considered statistically significant when *P* < 0.05. All statistical analysis was performed with SPSS for Windows, version 20.0 (SPSS Inc., Chicago, IL, USA).

## Additional Information

**How to cite this article**: Fukuo, M. *et al*. Screening for diabetic retinopathy using new mydriasis-free, full-field flicker ERG recording device. *Sci. Rep.* **6**, 36591; doi: 10.1038/srep36591 (2016).

**Publisher’s note:** Springer Nature remains neutral with regard to jurisdictional claims in published maps and institutional affiliations.

## Figures and Tables

**Table 1:** Demographic data of six groups.

	Normal	DM (+)					*P*-value
		No DR	NPDR			PDR	
			Mild	Moderate	Severe		
No. of eyes	48	74	11	13	10	10	
Age (yrs.)	52.7 ± 7.6	51.4 ± 9.7	56.4 ± 8.0	59.6 ± 6.7	53.4 ± 5.9	53.1 ± 6.3	0.10
Duration of DM (yrs.)		5.7 ± 4.4	8.0 ± 5.8	11.8 ± 4.2	13.1 ± 9.1	12.7 ± 7.7	0.001**
HbA1c (%)		7.9 ± 1.5	8.9 ± 1.2	8.7 ± 1.7	8.9 ± 1.4	8.2 ± 1.8	0.30
BCVA (log MAR)	−0.08 ± 0.05	−0.06 ± 0.07	−0.07 ± 0.04	0.03 ± 0.20	0.03 ± 0.08	0.14 ± 0.19	0.001**
Refractive error (D)	−1.3 ± 1.8	−1.7 ± 2.5	−1.8 ± 3.2	−2.4 ± 3.3	−2.0 ± 2.6	−2.5 ± 1.9	0.20

DM, diabetes mellitus; DR, diabetic retinopathy; NPDR, nonproliferative diabetic retinopathy; PDR, proliferative diabetic retinopathy; BCVA, best-corrected visual acuity; Log MAR, logarithmic minimum angle of resolution; D, diopter. Data are shown as the mean ± standard deviation. A one-way layout analysis of variance (ANOVA) was performed to examine the homogeneity of the background factors in each group. **P < 0.001.
